# Association between oral hypofunction and general health: a systematic review

**DOI:** 10.1186/s12903-023-03305-3

**Published:** 2023-08-24

**Authors:** Nareudee Limpuangthip, Orapin Komin

**Affiliations:** Department of Prosthodontics, Faculty of Dentistry, Chulalongkorn University, 34 Henri-Dunant Road, Pathumwan, Bangkok, 10330 Thailand

**Keywords:** Oral function, Mastication, Swallowing, Physical activity, Social activity, Cognitive impairment

## Abstract

**Background:**

Oral hypofunction comprises 7 oral function conditions, including mastication, swallowing, pronouncing, oral hygiene, and oral dryness. However, it remains unresolved whether having oral hypofunction and each individual condition adversely affect general health. This study aimed to investigate the association between oral hypofunction and general health, encompassing physical, psychological, and social dimensions.

**Methods:**

Oral hypofunction was diagnosed following the criteria proposed by the 2016 Japan Society of Gerodontology, comprising 7 oral function conditions. Oral hypofunction was diagnosed when at least 3 conditions presented. The selected studies eligible for reviewing were based on the PI(E)CO criteria: P (population), adult and older adults; I/E (intervention/exposure), having oral hypofunction by considering overall and individual 7 oral function conditions; C (comparator), not having oral hypofunction; O (outcome), the general health status, encompassing physical, psychological, and social dimensions. Electronic search limited to English articles was performed through PubMed, Scopus, and Cochrane Database of Systematic Reviews databases from January 2016 to June 2023. The included studies were assessed for quaility and risk of bias using the modified Newcastle-Ottawa scale. Qualitative analyses were performed on the general outcome, and having oral hypofunction considering overall and individual oral function conditions.

**Results:**

Ten studies were included in the final syntheses. One was a prospective cohort, one was a case-control, and the others were cross-sectional studies. All included studies were high quality. General health outcomes were mostly frailty, sarcopenia, and malnutrition, whereas mild cognitive impairment,social withdrawal, and gastric cancer prognosis were identified in one of the included studies. The association between general health and having oral hypofunction was stronger than when considering an individual condition. The health outcomes were predominantly associated with mastication and swallowing, whereas their relationships with oral hygiene and dryness were less likely to be reported.

**Conclusions:**

The present review found an association between the presence of oral hypofunction and impaired general health. However, the specific impact on individual physical, psychological, and social dimensions cannot be distinctly delineated. Further prospective studies are required to clarify the causal-effect relationship between oral and general health.

## Background

According to the FDI World Dental Federation definition, oral health encompasses the ability to perform daily activities, such as speaking and masticating, and maintaining usual emotions without pain, discomfort, and any diseases related to the craniofacial complex [1]. Poor oral health with declined oral function can have detrimental effects on overall health and mortality [2–4]. Oral health has been recognized as one of the essential components of overall health and well-being. Thus, early evaluation of oral function is necessary to prevent the deterioration of general health function at its initial stage.

Based on the 2016 diagnostic criteria proposed by the Japan Society of Gerodontology (JSG), oral function is divided into 4 stages from the least to the most severe: healthy, oral frailty, oral hypofunction, and dysfunction stages [5]. Oral frailty denotes a declined oral function, evident through the manifestation of signs and symptoms within the oral cavity. Meanwhile, oral hypofunction indicates pathophysiological functional conditions encompassing 7 impaired oral functions. Based on the Fujita Health University Hospital study [6], the results suggested that oral hypofunction requires the presence of at least 3 out of 7 oral function conditions, including mastication, swallowing, pronouncing, oral hygiene, and oral dryness. Oral hypofunction progression can result in irreversible dysfunction stage, characterized by eating, swallowing, and masticatory disorders. However, oral hypofunction can be recovered to the frailty and healthy stage by appropriate oral health care and dental treatment. Moreover, oral hypofunction escalates in conjunction with the deterioration of systemic function and impaired general health, such as malnutrition, frailty, cognitive decline, and social isolation [7–13].

**Fig. 1:**
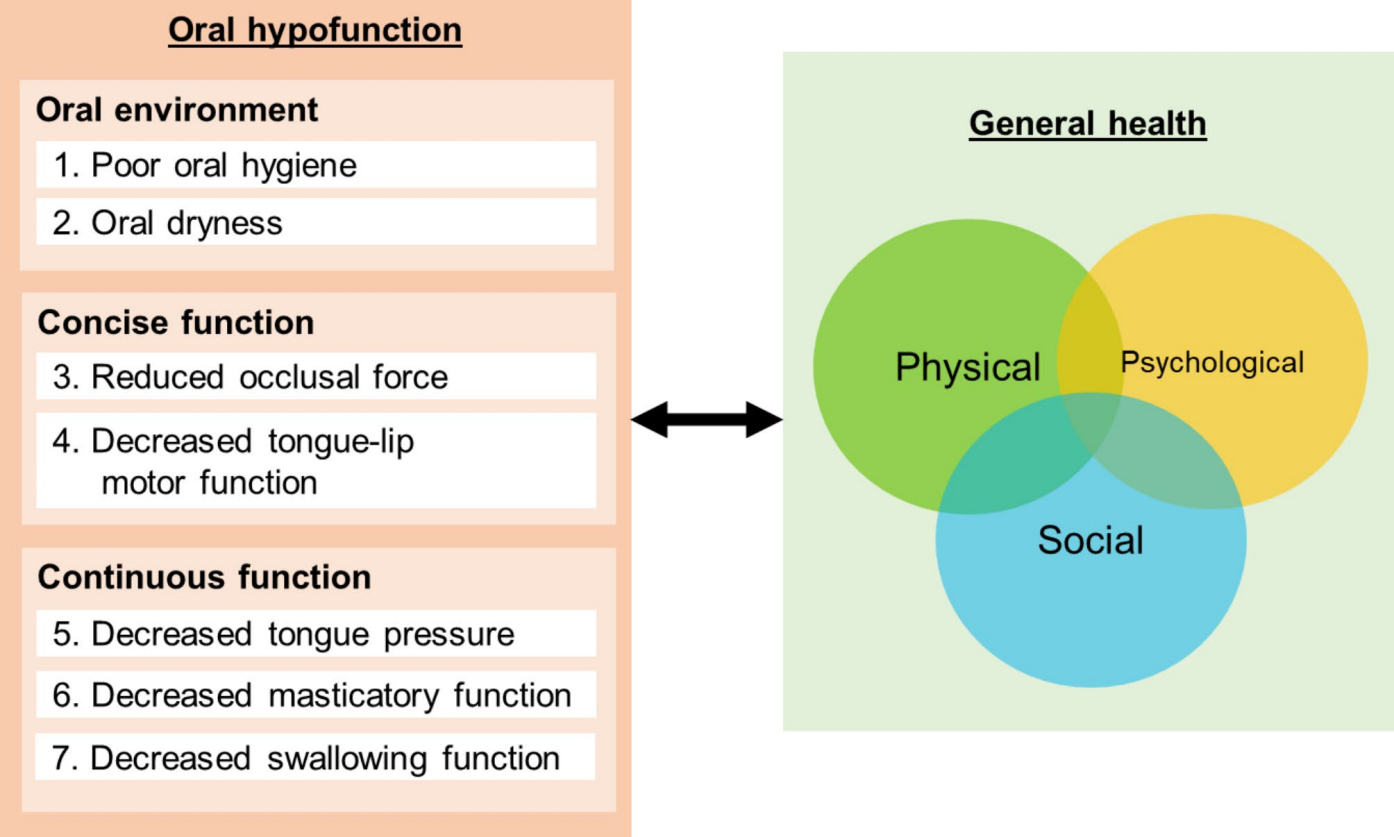
Conceptual framework of the study

In order to mitigate the declined general health, particularly in older individuals, the early diagnosis of oral hypofunction is essential. Furthermore, it would be valuable to ascertain which of the 7 conditions had more substantial impact on general health, as evaluating all conditions may be impractical in clinical settings where specific tools for assessing oral function are unavailable. General health encompasses more than just the absence of disease, but represents a state of complete physical, psychological, and social well-being, all of which are interconnected. However, whether having oral hypofunction and each individual condition adversely affect general health remains unresolved. Therefore, the aim of this systematic review was to investigate the association between oral hypofunction and general health, encompassing physical, psychological, and social dimensions. A conceptual framework of the possible links between the study variables is illustrated in Fig. 1.

## Methodology

This study was conducted following the Preferred Reporting Items for Systematic Reviews and Meta-Analysis (PRISMA) guidelines and statement [14]. The protocol for this systematic review was registered with PROSPERO (registration number CRD42023400194).

### Research questions

According to the World Health Organization (WHO), health is defined as a state of complete physical, mental, and social well-being [15]. Thus, the research question for this systematic review was “Is the presence of oral hypofunction associated with impaired general health, encompassing physical, psychological, and social dimensions?”

### Search strategy and study selection

An electronic search was performed using the PubMed, SCOPUS, and Cochrane Database of Systematic Reviews databases from January 2016 to June 2023. The search was limited to English-language articles and began in 2016 because the term oral hypofunction was first mentioned by the JSG in 2016. The literature search was performed by two independent investigators (N.L. and O.K.). The selected studies eligible for reviewing were based on the PI(E)CO criteria as follows: P (population), adult and older adults aged 18 years and above who could perform all 7 oral function tests; I/E (intervention/exposure), having oral hypofunction by considering overall and individual 7 oral function conditions; C (comparator), not having oral hypofunction by considering overall and individual oral function conditions; and O (outcome), general health status, encompassing the physical, psychological, and social dimensions. General health can be assessed through clinical examination or patient-reported outcome measures.

The search protocols included the following electronic keywords terms: (oral hypofunction) AND (health OR health status [MeSH] OR physical OR disease OR psychological OR psychological health OR mental OR social). In addition, a manual search for obtainable relevant studies using the references from the included articles was performed. Observational studies, including cross-sectional and longitudinal studies, conducted in general or specific population groups were included. The exclusion criteria were narrative and systematic reviews, editorial commentary, and case report. In addition, the articles that did not completely identify the 7 oral function conditions for oral hypofunction diagnosis were excluded [16–19].

### Data extraction

The titles and abstracts of each article were reviewed independently by two investigators and the articles that did not meet the PI(E)CO criteria were excluded. The full texts of the selected studies were read and the articles that did not meet the eligibility criteria were excluded. Any discrepancies were resolved by an external third reviewer who was a psychiatrist. The reviewers were not blinded regarding the articles’ authors in any stage.

A structured data collecting form was created using Microsoft Office Excel 2019 to record the information extracted from each study. The extracted information included the first author’s name, year of publication, characteristics of the study population, country, study design and follow-up duration, method of oral function evaluation and criteria for oral hypofunction diagnosis, the general health outcome, and potential confounders/covariates of the association between oral hypofunction and general health.

### Risk of bias assessment

The risk of each studies bias was assessed independently by the two investigators (N.L. and O.K.), and the psychiatrist. A modified Newcastle-Ottawa Scale was used as a criterion for quality assessment of the included studies [3, 20]. The score ranges from 0 to 7, the higher score indicates better quality of the study.

## Results

**Fig. 2:**
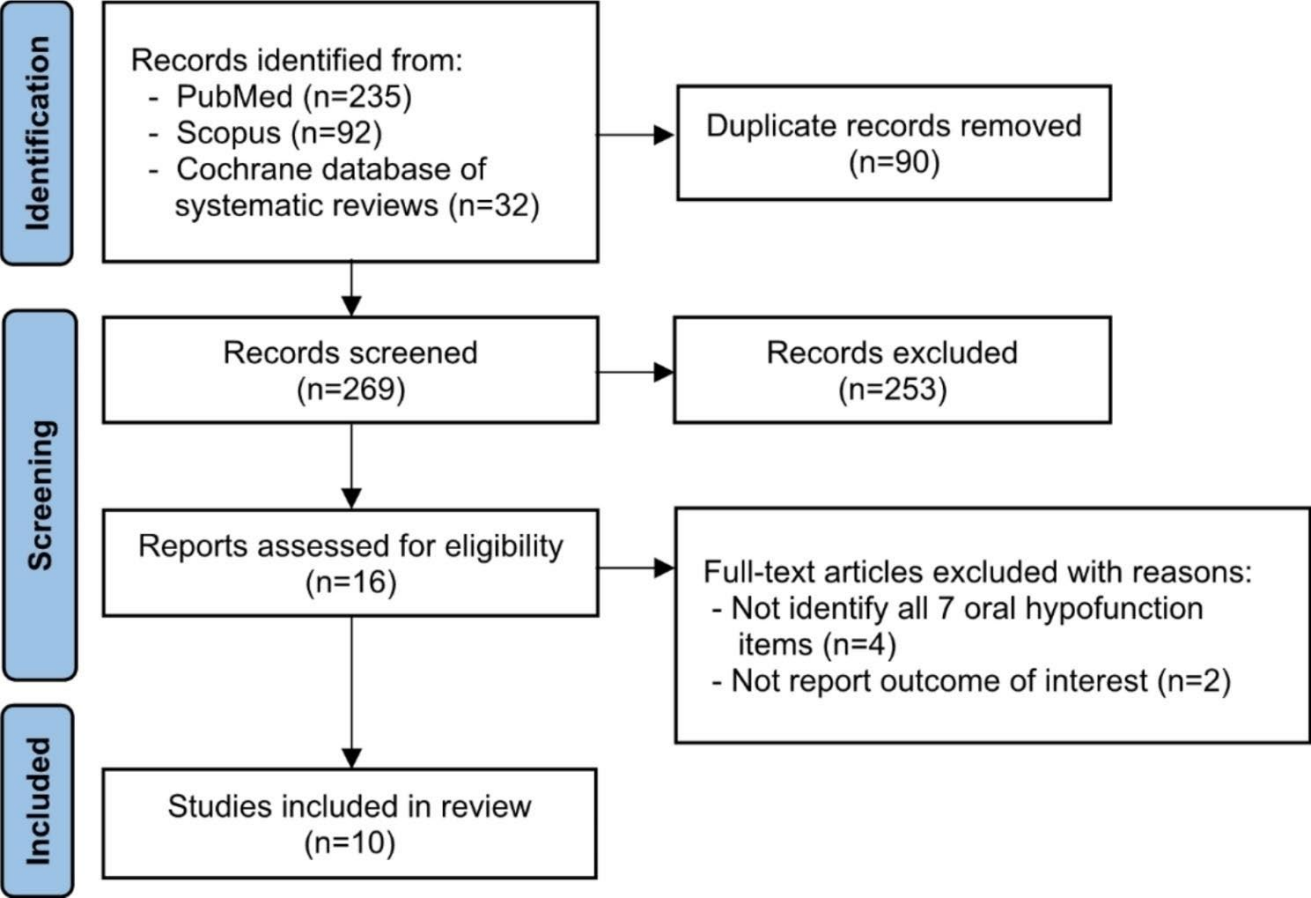
PRISMA flow diagram of the included studies

The PRISMA flow diagram is presented in Fig. 2. Table 1 presents the characteristics of the 10 included articles; one was conducted in older people in Switzerland, and the others were conducted in adults and older Japanese. The inclusion criteria were community-dwelling older adults [7–11, 13, 21], the patients who had regular dental maintenance [22], or primary gastric cancer patients [23]. The exclusion criteria of the participants varied among studies, including older adults who were unable to answer questions due to their inability to come to the examination site [9], were hospitalized or lived at a care facility [8], having cognitive decline or history of dementia [9, 11], having risks associated with physical assessment, such as history of cardiovascular disease and > 200 mmHg systolic blood pressure [13], or having conditions that affected oral intake, such as poorly-controlled diabetes and gastrointestinal symptoms [12]. Four included articles used information from the two same clinical settings, however, they evaluated different cohorts [8, 10, 11, 21]. All the included studies assessed the presence of oral hypofunction by considering both the overall oral hypofunction and the 7 specific oral function conditions. They also examined the relationships between oral hypofunction and various indicators of impaired general health, including frailty [8, 9, 13], sarcopenia [8, 13, 21, 22], malnutrition [10–12], cognitive impairment [8], social withdrawal [7], and poor gastric cancer prognosis [23]. Notably, the evaluation of frailty and malnutrition using the Kihon checklist and MNA-SF, respectively, provided insights into not only the physical aspects but also the psychological and social dimensions of health. The quality of the included studies is presented in Table 2.

**Table 1:** Characteristics of the included studies and oral hypofunction prevalence (%)

Author (year)	Participants				Study setting; Country	Study design (Duration)	Oral hypofunction prevalence (%)	Covariate adjustment (in multivariable analysis)
	Characteristics	N	Age (y): mean ± sd. (range)	M:F ratio				
Shimazaki et al. (2020) [9]	Independent community-dwelling older adults	978	median [IQR]: 73.0 [8.0] (65–85)	1:1.69	Higashiura, Aichi Prefecture, Japan	Cross-sectional (Sep–Oct 2018)	60.0	- Age - BMI - Systemic medical conditions
Hasegawa et al. (2020) [7]	Independent older adults	427 (Baseline = 976)	72.7 ± 5.7 (≥ 65)	1:1.83	FESTA study, Japan	Prospective cohort (Baseline and 2-y follow-up)	NM	- Cognitive function - Physical indicator and performances
Nakamura et al. (2021) [8]	Community-dwelling older adults	832	74.9 ± 6.3 (≥ 65)	1:1.69	2018 Tarumizu study health survey, Japan	Cross-sectional (June–Dec 2018)	56.3	- Age, sex, education - BMI, physical activity
Nishi et al. (2021) [11]	Community-dwelling adults and older people	1004	67.5 ± 11.3 (≥ 40)	1:1.63	2019 Tarumizu study health survey, Japan	Cross-sectional (June–Dec 2019)	46.7	- Age, sex, education, living condition - BMI, skeletal muscle index - Smoking, energy intake - Systemic medical conditions
Kugimiya et al. (2021) [21]	Community-dwelling older adults	878	76.5 ± 8.3 (≥ 65)	1:2.28	2018 Otassha study, Japan	Cross-sectional (2018)	50.5	- Age, gender, education, living condition - BMI, drinking, smoking habits - Depression, Cognitive function - Systemic medical conditions, etc.
Iwasaki et al. (2021) [10]	Community-dwelling older adults	715	73.5 ± 6.6 (65–91)	1:1.56	2018 Otassha study, Japan	Cross-sectional (2018)	42.7	- Age, gender, education, living condition - Social isolation, social support - Drinking and smoking, BMI, physical activity - Systemic medical conditions - Denture use
Yoshida et al. (2022) [13]	Community-dwelling older adults	340	75.0 ± 8.3 (≥ 65)	1:2.28	Kyoto Elderly Physical Fitness Measurement Project-Japan	Cross-sectional (June 22–23, 2019)	53.5	- Age, sex - BMI
Ohta et al. (2022) [12]	Non-acute hospitalized older adults	60	median [IQR]:82.0 [11] (70–96)	1:1.72	Department of rehabilitation and long-term care of the University Hospital of Geneva; Switzerland	Cross-sectional (Oct 2019–July 2020)	88.3	- Age, sex, education - BMI, Reason for hospitalization - Systemic medical conditions - Dental status
Shirahase et al. (2022) [22]	Patients who had regular dental maintenance	269	74.9 ± 6.50 (≥ 65)	1:1.02	Dental clinic, Japan	Cross-sectional (June 2019–March 2021	49.1	- Age, sex - BMI - Systemic medical conditions
Abe et al. (2023) [23]	Patients with primary gastric cancer	95	67.2 ± 13.2 (37–89)	1:1.67	Nagoya Ekisaikai Hospital	Case-control (Jan 2014–Dec 2021)	25.3	- Age, sex - Smoking, alcohol consumption - Number of present teeth, Eichner classification

BMI, body mass index; CHS, Cardiovascular Health Study; Frail Elderly in the Sasayama-Tamba Area (FESTA); NM, not mentioned

**Table 2:** Quality assessment of the studies based on the Modified Newcastle-Ottawa scale

Study	Selection (0–1)				Comparability* (0–2)		Outcome (0–1)		Total score (0–7)
	Representativeness of exposed cohort	Non-exposed cohort selection	Ascertainment of exposure		Based on the design or analysis		Outcome assessment	Adequacy of follow-up	
Shimazaki et al (2020) [9]	1	1	1		2		1	0	6
Hasegawa et al (2020) [7]	1	1	1		2		1	1	7
Nakamura et al (2021) [8]	1	1	1		2		1	0	6
Nishi et al (2021) [11]	1	1	1		2		1	0	6
Kugimiya et al (2021) [22]	1	1	1		2		1	0	6
Iwasaki et al (2021) [10]	1	1	1		2		1	0	6
Yoshida et al (2022) [13]	1	1	1		2		1	0	6
Ohta et al (2022) [12]	1	1	1		2		1	0	6
Shirahase et al (2022) [23]	1	1	1		2		1	0	6
Abe et al (2023) [24]	1	1	1		2		1	0	6

* 1 point when the exposed subjects were matched with non-exposed subjects, 2 points when the study also adjusted for covariates or confounders.

The associations between impaired general health and oral hypofunction are shown in Table 3. Seven condition of oral hypofunction was assessed, comprising poor oral hygiene, oral dryness, reduced occlusal force, decreased tongue-lip motor function, reduced tongue pressure, decreased masticatory function, and deteriorated swallowing function [5]. Oral hypofunction was diagnosed when 3 or more of these diagnostic criteria were met [8–13, 21, 22], except for Hasegawa et al. (2020), who diagnosed oral hypofunction when at least 1 condition was present [7]. Four studies also determined overall oral hypofunction by considering the number of oral function items [10–12, 22]. The association between impaired general health and overall oral hypofunction was more frequently reported than that with an individual condition [8, 9, 11]. The health outcomes were more frequently associated with occlusal force, tongue function, as well as masticatory and swallowing function, whereas their relationships with oral hygiene and oral dryness were less likely to be reported [8, 9, 11, 13, 21, 22] (Table 4).

**Table 3:** General health outcomes and their associations with oral hypofunction [✓univariate, ✓✓multivariable analysis, (✓) indirect association with overall hypofunction, ✗ no association]

Author (year)	General health outcome:				Oral hypofunction items							[8] Overall oral hypofunction
	Status	Criteria		[1] Poor oral hygiene		[2] Oral dryness	[3] Reduced OF	[4] Decreased tongue-lip motor function	[5] Decreased TP	[6] Decreased masticatory function	[7] Decreased swallowing function	
Shimazaki et al. (2020) [9]	Frailty status	KCL		✗^[1.1]^		✗^[2.1]^	✓^[3.2]^	✓	✗	✗^[6.1]^	✓^[7.1]^	✓✓^[8.1]^
Hasegawa et al. (2020) [7]	Social withdrawal at baseline and 2-y follow-up	Went out less than once a week, and went out less than last year (at baseline and 2-y follow-up)		✓^[1.3]^		✓^[2.1], [2.2]^	✓^[3.1]^	✗	✗	✓^[6.4]^	✓^[7.2]^	✓✓^[8.2]^ (baseline condition affect 2-y follow-up)
Nakamura et al. (2021) [8]	1) Frailty status	1) CHS		✗^[1.2]^		✗^[2.1]^	✓^[3.2]^	✗	✓	✓^[6.3]^	✓, ✓✓^[7.1]^	✓^[8.1]^
	2) Sarcopenia	2) AWGS2019		✗^[1.2]^		✗^[2.1]^	✗^[3.2]^	✗	✗	✗^[6.3]^	✗^[7.1]^	✓^[8.1]^
	3) MCI	3) NCGG-FAT		✗^[1.2]^		✗^[2.1]^	✓✓^[3.2]^	✗	✓✓	✗^[6.3]^	✗^[7.1]^	✓^[8.1]^
Nishi et al. (2021) [11]	Malnutrition (Protein intake)	BDHQ nutritional assessment		✗^[2.1]^		✗^[2.1]^	✓^[3.1]^	✓	✗	✗^[6.3]^	✗^[7.1]^	✓,✓✓^[8.1], [8,3]^
Kugimiya Y, et al. (2021) [21]	Sarcopenia	AWGS2019		✗^[1.2]^		✗^[2.1]^	✓^[3.1], [3.3]^	✓	✓	✓^[6.2]^	✓^[7.1]^	✓✓^[8.1]^
Iwasaki I, et al. (2021) [10]	Malnutrition	MNA-SF		(✓)^[1.2]^		(✓)^[2.1]^	(✓)^[3.1], [3.3]^	(✓)	(✓)	(✓) ^[6.2]^	(✓)^[7.1]^	✓, ✓✓^[8.1], [8.3]^
Yoshida et al. (2022) [13]	1) Sarcopenia	1) AWGS2019		✗^[1.2]^		✗^[2.1]^	✗^[3.2]^	✗	✗	✓^[6.1]^	✓^[7.1]^	✓✓
	2) Frailty status	2) KCL and CHS		(✓)^[1.2]^		✗^[2.1]^	✗^[3.2]^	✗	✓	✓^[6.1]^ (CHS)	✓^[7.1]^	✓✓^[8.1]^ (KCL)
Ohta et al. (2022) [12]	Malnutrition	1) MNA-SF 2) NRS 3) GLIM		✗^[1.1]^		✗^[2.1]^	✗^[3.2]^	✗	✗	✗^[6.1]^	✗^[7.1]^	✓^[8.3]^, ✗^[8.1]^
Shirahase et al. (2022) [22]	Sarcopenia	AWGS2019		✗^[1.2]^		✗^[2.1]^	✗^[3.1]^	✓	✓	✗^[6.1]^	✗^[7.1]^	✓^[8.3]^, ✗^[8.1]^
Abe et al. (2023) [23]	Gastric cancer prognosis	Prognostic nutritional index		✗^[1.2]^		✗^[2.1]^	✗^[3.3]^	✗	✓, ✓✓	✗^[6.1]^	✗^[7.1]^	✗^[8.1]^

Abbrev. AWGS2019, the 2019 Asian Working Group for Sarcopenia; BDHQ, Brief-Type Self-Administered Diet History Questionnaire; GLIM, Global Leadership Initiative on Malnutrition criteria; KCL, Kihon Checklist score; MCI, mild cognitive impairment; MNA-SF, Mini Nutritional Assessment-Short Form; NCGG-FAT, the National Center for Geriatrics and Gerontology-Functional Assessment Tool; NRS, Nutritional Risk Screening

Code: [1] **Poor oral hygiene**: [1.1] bac count > 10^6.5^ colony forming unit /ml, [1.2] tongue coating index (TCI) ≥ 50%, [1.3] 9-item oral assessment chart; [2] **Oral dryness**: [2.1] oral moisture < 27, [2.2] KCL assessment; [3] **Reduced occlusal force**: [3.1] remaining teeth < 20, [3.2] occlusal force (OF) < 500 N, [3.3] OF < 200 N; [4] **Decreased tongue-lip motor function**: <6 counts of /pa/,/ta/or/ka per sec (all included studies); [5] **Decreased tongue pressure (TP)**: maximum TP < 30 kPa (all included studies); [6] **decreased masticatory function**: [6.1] glucose conc.<100 mg/dL after gummy jelly chewing, [6.2] gummy jelly chewing score ≤ 2, [6.3] inability to chew tough food, [6.4] RSST, KCL assessment; [7] **Decreased swallowing function**: [7.1] EAT-10 score ≥ 3, [7.2] KCL assessment, [7.3] ≥ 1 ‘A’ response from Seirei questionnaire; [8] **Overall oral hypofunction**: [8.1] hypofunction when ≥ 3 conditions, [8.2] hypofunction when ≥ 1 condition, [8.3] increased number of item

**Table 4:** Proportion of the studies that demonstrated an association with a health outcome to the number of studies that evaluated each outcome

Oral hypofunction items		Health outcomes (number of studies)					
	**Sarcopenia (4)** [8, 13, 21, 22]		**Frailty (3)** [8, 9, 13]	**Malnutrition (3)** [10–12]	**Gastric cancer prognosis (1)** [23]	**MCI (1)** [8]	**Social withdrawal (1)** [7]
1. Poor oral hygiene	0/4		1/3	1/3	0/1	0/1	1/1
2. Oral dryness	0/4		0/3	1/3	0/1	0/1	1/1
3. Reduced occlusal force	1/4		2/3	2/3	0/1	1/1	1/1
4. Decreased tongue-lip motor	2/4		1/3	2/3	0/1	0/1	0/1
5. Decreased tongue pressure	2/4		2/3	1/3	1/1	1/1	0/1
6. Decreased masticatory function	2/4		2/3	1/3	0/1	0/1	1/1
7. Decreased swallowing function	2/4		3/3	1/3	0/1	0/1	1/1
Overall oral hypofunction	4/4		3/3	3/3	0/1	1/1	1/1

MCI, mild cognitive impairment

## Discussion

The present systematic review investigated the association between oral hypofunction and impaired general health, including frailty, sarcopenia, malnutrition, mild cognitive impairment, social withdrawal, and poor gastric cancer prognosis. Impaired general health in adult and older individuals was found to be related with having oral hypofunction. However, its relationship with an individual oral function condition varied among studies. General health indicators comprised clinical examination of the patients and self-reported general health, predominantly sarcopenia [8, 13, 21, 22], frailty [8, 9, 13], and nutritional status [10–12]. To assess sarcopenia, the 2019 Asian Working Group for Sarcopenia (AWGS) criteria was used [8, 13, 21]. Sarcopenia was diagnosed when having low muscle strength, low muscle mass, and/or low physical performance [24, 7, 8].The mild cognitive impairment was evaluated using the National Center for Geriatrics and Gerontology-Functional Assessment Tool comprising 4 domains of memory, visual motor speed, executive function, and processing speed [8, 25]. Frailty was assessed using the Cardiovascular Health Study (CHS) criteria [26] and the Kihon checklist [27]. The CHS criteria assessed physical frailty based on the phenotype model, consisting of weight loss, exhaustion, low physical activity, slow walking speed, and weakness [27].The Kihon checklist evaluates activities of daily living, physical strength, depressed mood, cognitive function, oral function, nutritional status, and social isolation/withdrawal [28]. Hasegawa et al. (2020) selected 2 questions from the KCL checklist to represent social withdrawal, comprising went out less than once a week, and went out less compared with the last year [7].

Nutritional status was assessed using the short-form mini-nutritional assessment (MNA-SF) [10, 12], the Global Leadership Initiative on Malnutrition (GLIM) criteria, and Nutritional Risk Screening (NRS) [12]. The MNA-SF comprises 6 items, i.e., decreased food intake, weight loss, impaired mobility, psychological stress, neuropsychological problems, and abnormal body mass index (BMI) [29]. The GLIM considers phenotypic (weight loss, BMI, and fat-free mass index) and etiologic criteria (reduced food intake, C-reactive protein level) [30]. In contrast, the NRS assesses nutritional status (i.e., weight loss, food intake) and disease severity (i.e., stress metabolism) [31]. Nishi et al. (2021) focused on protein intake obtained from the Brief-Type Self-Administered Diet History Questionnaire using the diet reference intake for Japanese [11, 32]. The findings demonstrate the interconnection of physical, psychological, and social dimension of health, as shown by the capacity of a single tool, such as Kihon checklist and MNA-SF, to evaluate various dimension of health simultaneously.

The associations between impaired general health and declined individual oral function have been reported, however, the results are inconsistent. Similar to previous studies, our findings indicated that masticatory and swallowing functions [7–10, 13, 21], as well as their related factors, such as the number of remaining teeth, bite force, and tongue pressure are commonly associated with general health [8, 10, 11, 21]. Previous cross-sectional studies in older adults consistently demonstrate the links between declined masticatory performance and swallowing function with malnutrition [33], decreased muscle and bone mass, and increased body fat [4].However, a recent systematic review revealed that the relationship between poor oral health and sarcopenia remains inconclusive [34]. This may be attribute to studies primarily evaluating individual oral function conditions rather than overall oral hypofunction. Based on our findings, the associations between general health and having oral hypofunction were more pronounced than when considering an individual condition [8, 9, 11], and a notable association was found with an increased number of conditions [10, 11]. Ohta et al. (2022) reported that the sensitivity of detecting malnutrition risk increased when the cut-off oral function conditions increased from 3 to 5 conditions [12]. Thus, a regular comprehensive evaluation of oral function is suggested to facilitate early detection of adverse general health conditions that may be undetected when determining a single condition.

Based on the findings, oral hypofunction can be perceived in three different aspects: oral environment (oral hygiene and dryness), concise function (occlusal force, tongue-lip motor function), and continuous function (tongue pressure, masticatory, and swallowing functions). The results showed that health outcomes were more frequently associated with concise and continuous functions, while the relationships with oral environment were less commonly reported. In situation with limited resources and testing equipment, such as when conducting all 7 oral hypofunction tests is not feasible, a potential approach could involve prioritizing the evaluation of concise and continuous tests. Possible mechanisms between oral hypofunction and impaired general health have been proposed. First, there is a nutritional pathway linking oral hypofunction with sarcopenia and frailty. Impaired oral function may lead to avoidance of hard and fibrous foods, resulting in nutrient-deficient diet [35] and malnutrition [12, 33, 36]. Older adults with fewer remaining teeth or oral hypofunction often exhibit lower masticatory muscle strength, lower BMI [8, 12, 21], and reduced muscle mass [4, 11], thereby increasing the risk of developing sarcopenia and frailty [11, 21]. Second, the occlusal tooth support, jaw position stability, and proprioception of the periodontal ligament plays a role in maintaining body balance during walking [37, 38], which is a frailty indicator. Third, oral hypofunction can be associated with psychological health, specifically cognitive function. Tooth loss, which impairs masticatory function, has been linked to cognitive impairment and dementia by negatively affect memory and learning [39]. Conversely, decreased motor function of the tongue-lip and masticatory muscles can be a consequence of declined brain and perioral muscle functions [5]. Lastly, oral function is linked to general health through an oral health-related quality of life. Poor oral function negatively impacts a person’s ability to perform physical, psychological, and social activities, leading to anxiety and social avoidance [40]. Overall, these connections highlight the importance of addressing oral hypofunction in relation to overall health and well-being.

In addition to oral hypofunction, some covariates have been reported to be associated with adverse general health. Females are more likely to have oral dryness, lower occlusal force, and lower masticatory function compared with males [9]. Increased age was also related to declined oral function, which increased the tendency of frailty, sarcopenia, and mild cognitive impairment [8, 21]. Patients with hypertension and stroke history were more likely to become pre-frail and frail [9]. Advanced age and systemic disease might increase the effect of oral hypofunction on decreasing general health. Thus, preserving functional teeth until older age is important to maintain optimal oral function.

The findings from this systematic review have clinical implications. Regular evaluation of oral function, especially in older people, may reduce the risk of declined general health and increased longevity. Oral exercises, such as tongue-strengthening, mouth-opening, and swallowing exercises, should be included in a daily routine to improve oral function [41, 42]. Establishing good oral hygiene is essential to prevent dental caries and periodontal diseases, which are major causes of tooth loss, that negatively affect masticatory and swallowing functions [10, 21, 43]. To replace missing teeth and restore optimal functional occlusion, good-quality dental prostheses with optimal denture occlusion are necessary. Importantly, oral health education and oral disease prevention programs are required to maintain optimal oral health and prevent the shared risk factors of non-communicable diseases [44].

Because oral and general health is linked, a comprehensive health care approach between dentists and other healthcare personnel is essential. While a dentist is responsible for dental prosthesis treatment, other healthcare personnel should have basic health and oral health literacy, such as oral hygiene care and dietary consultation. Oral health training programs in primary healthcare settings for dental hygienists and caregivers should be implemented to motivate home oral care and allow older people to realize the importance of preserving their natural teeth and maintain optimal oral function.

Some potential biases were noted in the included studies. There might be a selection bias because the participants of the included studies predominantly comprised independent older adults who might have health concerns and could come to the examination site on their own [10, 13]. Furthermore, those who could not respond to the questionnaire due to cognitive decline [9, 11], or were unable to perform physical activity tests were excluded [13]. These reasons may explain why the associations between some oral function conditions and general health outcome could not be found. In addition, the prevalence of oral hypofunction and impaired health condition of the studied population may be underestimated. Moreover, some information bias could be present when evaluating masticatory function and general health through a patient-reported outcome.

The present review has some limitations. Due to the nature of cross-sectional studies, a time-series association or causal relationship between oral hypofunction and frailty cannot be concluded from the findings. The clear distinction on oral hypofunction impacts on each physical, psychological, and social dimensions cannot be made since a single health evaluation tool, such as Kihon checklist and MNA-SF, may reflect all physical, psychological, and social dimensions. Nearly all the included studies collected data only in Japanese older adults with similar ethnic, social, and environmental backgrounds. Moreover, the concept of oral hypofunction was proposed by the JSG, and several instruments and indicators were mainly developed in Japan, including bite force measurement devices and a masticatory performance analyzer. Therefore, the generalizability of the findings might be limited. Because the methods of oral hypofunction and general health outcome evaluations varied among studies, a meta-analysis could not be performed. Furthermore, the results regarding the associations between individual oral hypofunction conditions and general health slightly varied among studies. Moreover, some possible confounding variables that could affect the outcomes were not evaluated, such as nutritional knowledge [10], and existing systemic health conditions.

Further studies are suggested to clarify the effects of oral hypofunction on general health in other countries with different living environments and sociodemographic backgrounds, and individuals with disease-specific conditions, such as dementia and heart disease. Longitudinal studies are required to confirm the true causal-effect relationship between oral and general health and to identify whether there is any intervention that can improve oral function. Because decreased oral function is affected by oral diseases, such as periodontitis and tooth loss, further information on oral health status is needed to explain the mechanisms between oral disease, oral function, and general health. A simplified protocol, such as a screening questionnaire, for the early detection of clinical symptoms and evaluation of oral hypofunction should be proposed for further community-based study. This protocol may also address the practicality issues by reducing the number of oral function conditions to be measured, as evaluating all 7 conditions may be challenging in certain settings.

## Conclusions

The present systematic review demonstrates the associations between oral hypofunction and impaired general health. However, the specific impact on individual physical, psychological, and social dimensions cannot be distinctly delineated. The association between general health and overall oral hypofunction was more pronounced than when considering an individual oral function condition. The health outcomes were predominantly linked to occlusal force, mastication, and swallowing functions, whereas their relationships with oral hygiene and dryness were less frequently reported.

## Data Availability

The datasets used and/or analyzed during the current study are available from the corresponding author on reasonable request.
